# Implementing maternal and newborn health quality of care standards in healthcare facilities to improve the adoption of respectful maternity care in Bangladesh, Ghana and Tanzania: a controlled before and after study

**DOI:** 10.1136/bmjgh-2023-012673

**Published:** 2023-11-14

**Authors:** Alexander Manu, Veronica Pingray, Sk Masum Billah, John Williams, Stella Kilima, Francis Yeji, Fatima Gohar, Priscilla Wobil, Farhana Karim, Projestine Muganyizi, Deus Mogela, Shams El Arifeen, Maya Vandenent, Ziaul Matin, Indeep Janda, Nabila Zaka, Tedbabe D Hailegebriel

**Affiliations:** 1 Epidemiology and Disease Control, University of Ghana School of Public Health, Accra, Ghana; 2 Maternal, Newborn and Adolescents Health, UNICEF HQ consultant, New York, New York, USA; 3 Department of Mother and Child Health Research, Institute for Clinical Effectiveness and Health Policy, Buenos Aires, Argentina; 4 Maternal and Child Health Division, ICDDRB, Dhaka, Bangladesh; 5 School of Public Health, The University of Sydney, Sydney, New South Wales, Australia; 6 Department of Clinical Sciences, Dodowa Health Research Centre, Ghana Health Service, Accra, Ghana; 7 Research Publication and Documentation Section, National Institute for Medical Research, Dar es Salaam, United Republic of Tanzania; 8 Planning, Policy, Monitoring, and Evaluation Division (PPMED), Ghana Health Service, HQ, Accra, Ghana; 9 Health Section, UNICEF Eastern and Southern Africa Regional Office, Nairobi, Kenya; 10 Health, UNICEF Ghana, Accra, Ghana; 11 Department of Obstetrics & Gynaecology, University of Dar es Salaam Mbeya College of Health and Allied Sciences (UDSM MCHAS), Mbeya, United Republic of Tanzania; 12 National Blood Transfusion Unit, Ministry of Health, Social Development, Gender, Elderly and Children, Dar es Salaam, United Republic of Tanzania; 13 MCHD, Icddr B, Dhaka, Bangladesh; 14 Health, UNICEF Bangladesh, Dhaka, Bangladesh; 15 Maternal, Newborn and Adolescents Health, UNICEF, New York, New York, USA; 16 Health, UNICEF Pakistan, Islamabad, Pakistan; 17 Health, UNICEF, New York, New York, USA

**Keywords:** Maternal health, Intervention study, Public Health, Obstetrics, Health services research

## Abstract

**Introduction:**

Many women worldwide cannot access respectful maternity care (RMC). We assessed the effect of implementing maternal and newborn health (MNH) quality of care standards on RMC measures.

**Methods:**

We used a facility-based controlled before and after design in 43 healthcare facilities in Bangladesh, Ghana and Tanzania. Interviews with women and health workers and observations of labour and childbirth were used for data collection. We estimated difference-in-differences to compare changes in RMC measures over time between groups.

**Results:**

1827 women and 818 health workers were interviewed, and 1512 observations were performed. In Bangladesh, MNH quality of care standards reduced physical abuse (DiD −5.2;−9.0 to –1.4). The standards increased RMC training (DiD 59.0; 33.4 to 84.6) and the availability of policies and procedures for both addressing patient concerns (DiD 46.0; 4.7 to 87.4) and identifying/reporting abuse (DiD 45.9; 19.9 to 71.8). The control facilities showed greater improvements in communicating the delivery plan (DiD −33.8; –62.9 to –4.6). Other measures improved in both groups, except for satisfaction with hygiene. In Ghana, the intervention improved women’s experiences. Providers allowed women to ask questions and express concerns (DiD 37.5; 5.9 to 69.0), considered concerns (DiD 14.9; 4.9 to 24.9), reduced verbal abuse (DiD −8.0; −12.1 to –3.8) and physical abuse (DiD −5.2; −11.4 to –0.9). More women reported they would choose the facility for another delivery (DiD 17.5; 5.5 to 29.4). In Tanzania, women in the intervention facilities reported improvements in privacy (DiD 24.2; 0.2 to 48.3). No other significant differences were observed due to improvements in both groups.

**Conclusion:**

Institutionalising care standards and creating an enabling environment for quality MNH care is feasible in low and middle-income countries and may facilitate the adoption of RMC.

**WHAT IS ALREADY KNOWN ON THIS TOPIC**

- Although respectful maternity care (RMC) is a crucial component of quality of care and a human right, there is evidence that many women do not access RMC, particularly in low-resource settings.
- A large body of evidence describes the extent of RMC, and very few studies have evaluated interventions with robust methodologies to address RMC.

**WHAT THIS STUDY ADDS**

- We performed a multicountry comparative before-and-after evaluation to measure the effect of implementing the maternal and newborn health (MNH) quality of care standards on RMC measures.
- Implementing MNH quality standards under real-world health system conditions was associated with improvements in effective communication, respectful and dignified care measures and women’s satisfaction. In addition, it improved some contextual factors, enabling environments to support changes and improvements.

**HOW THIS STUDY MIGHT AFFECT RESEARCH, PRACTICE OR POLICY**

- This study suggests that healthcare facilities and systems in low and middle-income countries can accelerate RMC by implementing MNH quality standards and developing multilevel, context-specific interventions when adequate investment and support are provided.

## Introduction

The current global agenda focus on the survival of women and their babies during childbirth and ensuring that they thrive and realise their full potential.^1^ Respectful maternity care (RMC) is a human right-based approach that can improve women’s pregnancy, labour and childbirth experience and address health inequalities.^1^ RMC refers to care organised for all women and provided to them in a manner that maintains their dignity, privacy and confidentiality; ensures freedom from harm and mistreatment and enables informed choice and continuous support during labour and childbirth.^2^ However, many women, particularly those in low and middle-income countries (LMICs), cannot access RMC.^3^ Many women experience poor quality of care (QoC) and treatment during childbirth, including disrespect and violations of their rights to privacy, informed consent and having a companion of choice during childbirth.^3–6^ These negative experiences of care can prevent women from seeking care in facilities during the postnatal period and for their subsequent deliveries.^4^ Additionally, disrespectful, abusive or neglectful care during childbirth may have direct adverse consequences for both the mother and infant.^7^

Women place a high value on RMC, and most healthcare providers would like to provide respectful, dignified and woman-centred care but may feel unable to do so due to resource constraints.^8^ Most research studies focus on identifying the extent and nature of gaps in providing RMC, and very few evaluate interventions to improve RMC. The latter often focus on training providers and fail to demonstrate a consistent sustained change over time.^9–13^ On the other hand, the literature suggests that complex, context-specific interventions targeting multiple levels of the health system are most likely to be effective in improving RMC.^8 9 11 14–18^ There is a need to advance from understanding the nature and extent of RMC gaps to developing and evaluating interventions designed to improve and sustain the adoption of RMC.^9^

In 2016, UNICEF/WHO published maternal and newborn health (MNH) QoC standards to improve the quality of maternal and newborn care, address health system inequities and strengthen accountability. The nine standards focused on providing evidence-based, safe care; experiencing dignified and respectful care for women and newborns and creating an enabling environment for such care.^19^ The standards envisioned experience of care in three domains: (a) effective communication, (b) social and emotional support and (c) respectful and dignified care. Although guidance to improve maternal and newborn care by implementing quality standards has been developed, no study has targeted improving RMC through a standard-based MNH QoC improvement pathway.^20^ The implementation of these standards was evaluated in seven intervention districts in Bangladesh, Ghana and Tanzania to inform the feasibility and effect of their institutionalisation within health systems. We present an evaluation of the effect of implementing the MNH QoC standards on RMC measures, focusing on effective communication, emotional support, respectful and dignified care and maternal satisfaction in each country.

## Methods

### Study design

**Figure 1:**
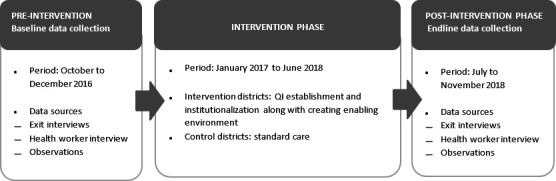
Timeline for the evaluation of the effect of implementing MNH quality of care standards implementation in healthcare facilities.

We used a facility-based, controlled before and after design to measure the effect of implementing the MNH QoC standards on RMC measures. The evaluation was conducted in seven intervention districts in Bangladesh, Ghana and Tanzania. Eight adjoining districts with similar characteristics as the intervention districts were evaluated for comparison. Data were collected during two time periods: between October and December 2016 (baseline) and 18 months later, between July and November 2018 (endline) (figure 1). Multiple data collection methods were used: interviews with health workers (HWs), exit interviews with women and observation of woman–provider interaction during and after labour and childbirth. Standards for Quality Improvement Reporting Excellence (SQUIRE) guidelines were used for reporting the results.^21^

### Setting and participants

**Figure 2:**
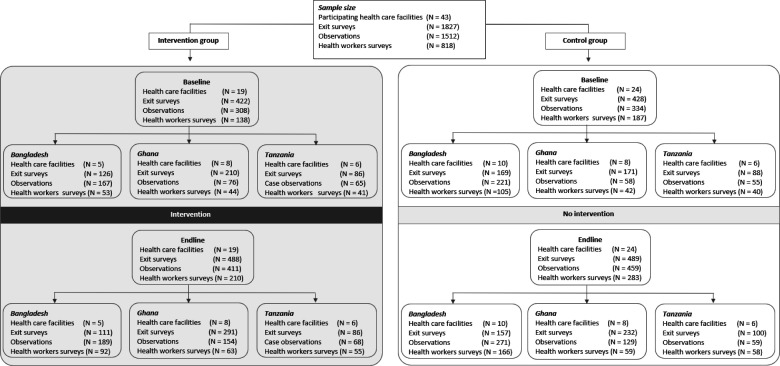
Participating healthcare facilities, women enrolled, health workers interviewed, and woman-provider interaction observations organised by group and country.

Participating regions (the Rangpur Region of Bangladesh, the Upper East region of Ghana and the Njombe region of Tanzania) were purposefully selected and prioritised by the ministries of health (MOH) of each respective country. Box 1 describes the context of maternity care in each country. Facilities were selected from the public sector based on their designation to provide emergency obstetric and newborn care, maternity caseload, the absence of quality improvement (QI) interventions at baseline and catchment populations’ sociocultural case mix (online supplemental table S1). A total of 43 health facilities were included: 15 in Bangladesh, 16 in Ghana and 12 in Tanzania. Nineteen intervention facilities were selected to implement the NMH QoC standards. Individual characteristics of each healthcare facility, including the type of model of care, are described in online supplemental table S2. The intervention was assigned to UNICEF-focused districts based on criteria such as low coverage of health interventions (in Kurigram, Bangladesh), social disadvantages (in Bawku Municipal, Bolgatanga Municipal, Bongo District, Kassena Nankana and West District, Ghana) or poor MNH indicators (Ludewa and Wanging'ombe, Tanzania). Twenty-four control facilities were selected from adjoining districts with populations of similar demographic characteristics. Figure 2 describes participating healthcare facilities, women enrolled, HWs interviewed and woman–provider interaction observations organised by group, time of evaluation (baseline; endline) and country. The population of the intervention districts was approximately 2.8 million.^22–24^

10.1136/bmjgh-2023-012673.supp1 Supplementary data

Box 1 **Context of maternity care in Bangladesh, Ghana and Tanzania**

**Bangladesh**

- The evaluation was conducted in the Ragpur region, which has a population of 2 069 273.
- In 2016, the region had a maternal mortality ratio of 222 deaths per 100 000 live births and 37 neonatal deaths per 1000 live births.
- The region has 69 hospitals and 2541 health centres, with 46% of births occurring in health facilities.
- Kurigram district was selected to implement the intervention due to low coverage of health interventions, while Gaibandha and Lalmonirhat districts were selected as controls.

**Ghana**

- The assessment was conducted in the Upper East Region, which has a population of 1 109 338.
- In 2016, the maternal and neonatal mortality rates varied depending on the data source. According to the District Health Information Management System, there were 111 maternal deaths per 100 000 live births and seven neonatal deaths per 1000 live births. However, the 2017 Maternal Health Survey reported a mortality risk of 310 maternal deaths per 100 000 live births and 24 neonatal deaths per 1000 live births based on survey data collected in 2014 (Ghana Demographic and Health Survey (GDHS) 2014).
- There are 164 healthcare facilities in the region and 1118 Community-Based Health Planning and Services, with 70% of births occurring in health facilities.
- Due to social disadvantages, Bawku Municipal, Bolgatanga Municipal, Bongo District, Kassena Nankana and West District were selected to implement the intervention. In contrast, Builsa North District, Kassena Nanakana Municipal, Bawku West District, Talensi District, Gaibandha and Lalmonirhat districts were selected as controls.

**Tanzania**

- The evaluation was conducted in the Njombe region, which has a population of 803 299.
- In 2016, the region had a maternal mortality ratio of 101 deaths per 100 000 live births and 31 neonatal deaths per 1000 live births.
- The region has 10 hospitals and 263 health centres, with 87% of births occurring in health facilities.
- Ludewa and Wanging’ombe districts were selected to implement the intervention due to poor maternal and newborn health indicators, while Njombe and Makete districts were selected as controls.

### Intervention

The intervention combined several strategies proposed to implement the MNH QoC standards based on QI frameworks that have been shown to change providers’ behaviours,^25 26^ institutionalise quality caregiving^27^ and address maternal and newborn care quality gaps.^19 28^ The main strategies used in the intervention group consisted of implementing the Every Mother Every Newborn QoC standards by (a) establishing and institutionalising QI teams and processes with the involvement of all-level leadership (MOH, district, facility and unit leaders) and (b) creating an enabling environment (including the development of infrastructure) to support the provision of quality care for mothers and newborns. Specific interventions were implemented as part of efforts to improve the care provided and experienced by mothers and caregivers. These included specific training of clinical staff in the intervention facilities on caregiving with compassion and respect as part of training on other QI interventions. Healthcare providers were also trained to engage women and their families in the decisions around the care they experienced, including seeking consent for interventions. Training also covered solicitation of client feedback and using the feedback to improve performance and quality.

In addition, to establish and institutionalise QI processes, QI teams defined change ideas, set objectives around these and implement them within Plan‒Do‒Study‒Act cycles. They use data for decision-making on quality and monitoring improvements.

Creating an enabling environment involved instituting structural changes to ensure better privacy, although temporary. In the case of the latter, some facilities procured curtains to provide separate enclosures for women in labour as part of ensuring visual privacy. Water, sanitation and hygiene as well as overall infection prevention and control measures, were identified as quality issues that affect client experience of care and were emphasised. For instance, it was considered that the cleanliness of the dedicated toilet for women within the maternity unit was a critical component of the experience of respectful care. Creating an enabling environment (eg, physical resources, human resources, policies, guidelines) to support quality care for mothers and newborns was also a key.

The intervention was pragmatically implemented in the context of routine intrapartum. Health facilities in the control group continued usual intrapartum care practice without introducing the MNH QoC standards.

### Measures and data collection

RMC was measured along the domains included in the WHO framework to assess the experience of care in the context of healthcare services—effective communication, emotional support and respectful and dignified care—from the perspectives of women, HWs and observers.^19^ These were supplemented with input measures (eg, physical resources, human resources, policies, guidelines).^29^ Independent clinicians and social scientists were trained for data collection at a 7-day workshop facilitated by the study coordination. Data were collected in each facility with piloted standardised structured paper-based forms, translated to local languages and culturally adapted. Data collectors obtained consent and conducted 1827 exit interviews, interviewed 818 health personnel and directly observed 1512 woman–provider interactions. Given the consecutive invitation of all women, sometimes the same woman who participated in the observation participated in the exit interview. However, this was not a criterion for inclusion in the exit interview, and some women participated only in the observation or only in the exit interview.

Below is a summary of the standardised procedures for measurement and data collection, while detailed information has been published elsewhere.^30^

#### Exit interviews

All postpartum women recently discharged from the postnatal ward were invited to participate, regardless of age and perinatal outcome. Interviews were conducted in private rooms. The questionnaires included sections that explored women’s sociodemographic characteristics and their perceived experience of care during and around intrapartum care, including privacy, HWs’ attitudes, communication, responsiveness to women needs, respect for women’s preferences, satisfaction and experiences of disrespect and abuse.

#### HW interview

HWs providing care during labour and childbirth (specialists, doctors, midwives and staff nurses) were invited to participate in structured interviews to explore contextual factors potentially influencing practice change, such as formal RMC training, policies and procedures for addressing patients’ concerns, rights and identifying and reporting abuse.

#### Clinical observations

All women consecutively admitted for labour and childbirth during the data collection visits were invited to participate in the woman–provider interaction observation. The observation was initiated at reception and lasted until the immediate postnatal period. Shifts of independent, external observers covered 24 hours a day and 7 days a week. Each observer stayed with the same woman during the whole process. Data collectors used a structured observation checklist to assess communication between HWs and women, privacy, supportive care, labour and delivery ward layout, occupancy and availability of human resources in health.

### Data management and statistical analysis

Data were collected in paper forms and entered into password-protected servers in each country. Study investigators conducted data quality assurance through supportive supervision with biweekly field visits to physically verify the completeness, accuracy and consistency of the data.

The analysis focused on reported and observed measures of RMC. It included several measures for each experience of care domain—effective communication, emotional support and respectful and dignified care. In addition, the experience of care measures was assessed by measuring overall satisfaction with care, HWs’ attitudes, overall hygiene, desire to return to the same health facility and recommendation of the facility to relatives/friends. Finally, we measured selected contextual factors that might enable or inhibit practice change.

We described women, HWs’ and observations’ baseline characteristics in each country using proportions to summarise categorical data, mean and SD or median and IQR according to data distribution for continuous data.

We compared temporal changes in RMC measures (measured as proportions with the exception of the measure ‘Number of HWs on duty’, which was measured on a numerical scale and summarised with means and SD) between the two groups using the difference-in-difference (DiD) analytical approach with models that included the main effects of group, time of evaluation and a two-way interaction term, separately for each country, adjusting for the cluster (facility) effect with robust SEs to correct for heteroscedasticity. This analytical method is used in quasiexperimental (nonrandomised) designs, where the two groups do not start at the same level at baseline. The DiD is implemented by computing two differences between groups: the first is the difference in the outcome variable between the two periods for each group. The second difference is the difference between the differences calculated for the two groups. The DiD estimate represents the differential improvements or declines in the outcomes of interest associated with the intervention. Significance was set at p=0.05, and 95% CIs were reported around estimates. Before conducting the analysis, we compared the characteristics of the groups at baseline and endline and did not find any substantial difference in participants’ characteristics (online supplemental table S4). The composition of the intervention and comparison groups was stable over time. In addition, the outcomes did not determine the selection of intervention districts. Intervention facilities were selected because the local government identified them as being located in areas with socioeconomic disadvantages and poor health indicators. Finally, we assumed that the intervention group’s outcomes would slowly improve (have a parallel trend), similar to the control group, given that RMC is on the international and national agendas. Stata V.14 (StataCorp, College Station, Texas) was used for the analyses.

### Patient and public involvement

The data collection instruments were pretested in all three countries to assess their acceptability to women and to adapt them culturally based on their suggestions. We interviewed women and family members in all participating facilities to obtain their perspectives on the care they received during labour and delivery. Special efforts were made to ensure confidentiality by storing all paper forms in locked cabinets with face sheets separated from study forms; electronic data were deidentified using participant ID, and no other identifiers were included in the data set. Women and family members are thanked for their contributions in the acknowledgements of this publication.

## Results

### Characteristics of women, HWs and observations

Women who participated in the exit interviews were very similar in both groups in all three countries except for Bangladesh, where at baseline, more adolescents (32.5% vs 17.8%) and women with lower education levels were in the intervention group than in the control group (24.6% of women completed middle/high school vs 32.5%) (table 1).

**Table 1:** Baseline characteristics of participating women, health workers and observations by group and country

Variables	Bangladesh		Ghana		Tanzania	
	Intervention	Control	Intervention	Control	Intervention	Control
	%	%	%	%	%	%
**Interviewed women**	**N=126**	**N=169**	**N=210**	**N=171**	**N=86**	**N=88**
Age in years						
Less than 20	32.5	17.8	14.3	12.3	17.6	17.1
20–34	60.3	76.3	71.9	72.5	69.4	73.9
35 o more	7.1	5.9	13.8	15.2	12.9	9.1
Education level						
Primary incomplete/no schooling	31.0	30.8	34.3	32.8	5.9	12.5
Primary complete	44.4	36.6	25.2	25.2	68.2	69.3
Middle/secondary	24.6	32.5	35.2	38.6	18.8	17.1
Post-secondary education	NA	NA	5.2	3.5	7.1	1.1
Number of deliveries (including this one)						
1	50.8	53.9	32.4	30.4	42.6	39.8
2–3	42.1	39.1	47.6	42.7	36.5	42.1
4 or more	7.1	7.1	20.0	26.9	21.2	18.2
**Interviewed health workers**	**N=53**	**N=105**	**N=44**	**N=42**	**N=41**	**N=40**
Age in years						
<30	20.8	16.2	40.9	45.2	26.8	45.0
30 and<40	34.0	47.6	29.5	26.2	36.6	27.5
≥40	45.3	36.2	29.5	28.6	36.6	27.5
Professional role						
Nurse	47.2	63.8	18.2	14.3	29.3	25.0
Midwife	3.8	1.9	65.9	73.8	43.9	30.0
Doctor	32.1	26.7	4.5	7.1	17.1	27.5
Other	17.0	7.6	11.4	4.8	9.8	17.5
Number of years in current position						
≤5	67.9	57.2	25.0	16.7	87.8	75.0
>5–10	5.7	9.5	9.1	16.7	4.9	10.0
>10	26.4	33.3	65.9	66.7	7.3	15.0
Gender						
Female	66.0	77.1	88.6	81.0	78.1	65.0
Male	34.0	22.9	11.4	19.0	21.9	35.0
**Observations during labour and childbirth**	**n=167**	**n=221**	**n=76**	**n=58**	**n=65**	**n=55**
Level of healthcare facility						
Health centre	53.9	58.6	14.5	17.2	27.7	41.8
Hospital	46.1	41.4	85.5	82.8	72.3	58.2
Day of the week						
Monday to Friday	74.7	71.0	65.8	75.9	67.7	72.7
Saturday or Sunday	25.3	29.0	34.2	25.1	32.3	27.3
Moment of arrival						
Day	66.1	60.5	47.4	46.5	64.6	50.9
Night	33.6	37.4	43.4	41.4	35.4	49.1
Missing	0	0	9.2	12.1	0	–
Maternal age in years						
Less than 20	18.6	17.6	9.2	10.3	18.5	12.7
20–34	77.3	76.6	63.2	56.9	67.7	72.7
35 or more	4.2	5	15.8	25.9	0	0

Bangladesh did not measure postsecondary education as a separate category; the highest education level category was intermediate/secondary or higher.

Interviewed HWs were similar in both groups at baseline, with a few exceptions. In Bangladesh, in the intervention group, HWs were slightly older (45.3% were ≥40 years old vs 36.2% in the control group), and there were fewer nurses compared with other healthcare cadres (47.2% vs 63.8% in the control group). In Tanzania, the intervention group had slightly fewer young HWs (26.8% were <30 years old vs 45.0% in the control group), more midwives (43.9% vs 30%) and fewer physicians (17.1% vs 27.5%). There were no differences between the intervention and control groups in Ghana, but compared with other countries, HWs were younger, more experienced, and the majority were midwives (65.9%).

The observations across all countries had similar baseline characteristics regarding the type of facility, day of the week, time of arrival (day/night) and woman’s age. A baseline difference was identified in Tanzania, where there were slightly more observations performed in hospitals than in health centres in the intervention group compared with the control group (72.3% vs 58.2%).

### RMC measures

Table 2 describes the relative frequencies of each RMC measure for each group (intervention and control), time of evaluation (preintervention and postintervention) and country. Table 3 reports DiD estimates for each RMC measure and country.

**Table 2:** Frequency of RMC measures by group, time of evaluation and country

RMC measure	Bangladesh				Ghana				Tanzania			
	Pre-intervention		Post-intervention		Pre-intervention		Post-intervention		Pre-intervention		Post-intervention	
	Intervention	Control	Intervention	Control	Intervention	Control	Intervention	Control	Intervention	Control	Intervention	Control
	%	%	%	%	%	%	%	%	%	%	%	%
**Woman exit interview**	**N=126**	**N=169**	**N=111**	**N=157**	**N=210**	**N=171**	**N=291**	**N=232**	**N=86**	**N=88**	**N=86**	**N=100**
Effective communication												
HW informed the woman of the findings	56.4	42.6	75.0	71.2	39.2	45.1	46.6	41.4	58.8	60.2	76.7	71.0
Communication was friendly	65.9	30.8	75.0	51.3	91.9	98.3	89.4	91.7	65.9	87.5	89.6	91.0
Enabled to ask questions/concerns	76.2	67.1	96.4	87.8	75.7	88.9	92.1	67.8	74.1	78.4	84.7	74.4
Emotional support												
HW took woman’s concerns into consideration	85.5	71.9	98.2	91.0	87.1	99.4	95.5	92.6	88.1	90.7	80.2	84.0
HW was responsive when the woman asked for support	91.3	86.9	100.0	90.4	93.3	99.4	97.3	97.8	90.6	95.5	97.7	99.0
Respectful and dignified care												
Privacy was ensured	86.5	59.8	96.4	76.9	89.5	98.3	94.5	95.2	71.8	94.3	97.7	96.0
The woman felt treated with respect	90.5	89.9	100.0	96.2	94.3	99.4	97.3	96.7	94.1	100.0	98.9	100.0
Verbal abuse	8.7	4.7	0.9	5.1	9.5	2.9	3.4	4.8	7.1	2.3	2.3	1.0
Physical abuse	3.2	1.2	0	3.2	3.8	0.6	1.0	3.0	1.2	1.1	1.2	1.0
Sexual abuse	0.8	0.6	0	0	1.0	0.0	1.4	1.3	0.0	1.1	1.2	1.0
Overall satisfaction												
Very satisfied with the care	27.0	14.2	47.6	33.6	27.3	55.6	66.9	57.8	9.4	50.0	38.4	62.0
Will come for another delivery	62.7	28.9	85.6	58.7	73.2	88.9	80.5	78.7	64.7	80.7	91.9	89.0
Would recommend this facility	94.4	97.4	99.1	93.6	92.8	97.1	95.5	97.4	88.2	94.3	100.0	97.0
Satisfied with the attitude of the health worker(s)	89.7	94.6	100.0	93.6	89.5	96.5	98.3	96.5	89.4	88.6	98.8	100.0
Satisfied with overall hygiene	10.3	8.3	15.3	16.6	66.9	92.1	91.9	94.6	10.6	46.6	25.6	52.0
**External nonparticipant observation**	**N=167**	**N=221**	**N=189**	**N=271**	**N=76**	**N=58**	**N=154**	**N=129**	**N=65**	**N=55**	**N=68**	**N=59**
Effective communication												
HW informs the woman of findings	52.7	46.7	94.5	77.9	69.7	75.4	96.7	94.6	67.7	54.5	77.6	74.1
HW informs the delivery plan	86.3	60.4	61.8	69.6	52.6	66.1	96.1	83.1	83.1	58.2	70.7	67.2
Courteous communication between HW and the woman	56.9	65.4	66.3	58.3	92.1	85.7	99.2	92.4	92.3	87.5	98.3	91.4
Emotional support												
HW is caring and supportive when the woman is in pain	59.3	42.8	59.0	47.8	84.0	78.6	89.5	88.4	89.2	67.3	94.3	90.6
HW refuses a request made (if requested)	0.9	6.0	0	8.7	4.0	1.2	2.3	0	2.7	8.8	8.0	4.2
Respectful and dignified care												
Privacy was ensured during the initial examination	43.1	29.3	60.7	38.2	10.5	35.1	55.8	70.8	81.5	83.6	87.9	77.6
Privacy ensured during labour	38.5	36.1	49.7	29.5	14.7	23.2	54.9	70.5	75.4	85.5	84.9	86.8
Privacy ensured during delivery	0.7	0.6	0	2.6	28.4	47.3	68.7	92.9	93.6	90.2	93.9	88.1

Denominators vary for each measure because the same sections were not observed in all women, as some women were admitted due to complications, needed caesarean section, or were admitted in the second stage of labour.

HW, health worker; RMC, respectful maternity care.

**Table 3:** Difference-in-difference estimates for each RMC measure by source of information and country

RMC measures	Bangladesh	Ghana	Tanzania
	Difference-in-difference (95% CI)		
**Woman exit interview**			
Effective communication			
HW informed the woman of findings	–9.8 (–69.9 to 50.1)	11.0 (–8.2 to 30.3)	7.2 (–21.7 to 36.0)
Communication was friendly	–11.4 (–48.5 to 26.5)	4.0 (–1.9 to 9.9)	20.2 (–16.3 to 56.6)
HW enabled to ask questions and express concerns	–0.5 (–13.3 to 12.2)	37.5* (5.9 to 69.0)	14.6 (–27.1 to 56.4)
Emotional support			
HW took woman’s concerns into consideration	–6.4 (–15.11 to 8.1)	**14.9**† (4.9 to 24.9)	–1.1 (–21.3 to 19.2)
HW was responsive when the woman asked for support	5.3 (–10.3 to 21.0)	5.5* (0.7 to 10.3)	3.5 (–8.4 to 15.2)
Respectful and dignified care			
Privacy was ensured	–7.2 (–54.5 to 40.0)	8.0* (0.6 to 16.0)	24.2* (0.2 to 48.3)
The woman felt treated with respect	3.3 (–10.2 to 16.8)	5.5* (1.0 to 9.9)	3.6 (–1.9 to 9.0)
Verbal abuse	–8.2 (–16.6 to 0.09)	**–8.0**† (–12.1 to –3.8)	–3.5 (–14.9 to 8.1)
Physical abuse	**–5.2‡** (−9.0 to –1.4)	–5.2* (–11.4 to –0.9)	0.1 (–5.1 to 5.4)
Sexual abuse	–0.2 (–2.3 to 1.9)	–0.8 (–3.4 to 1.7)	1.3 (–28.4 to 54.4)
Overall satisfaction			
Very satisfied with the care	1.4 (–35.7 to 38.5)	37.4 (–19.2 to 93.8)	17.0 (–12.8 to 46.7)
Will come for another delivery	–6.8 (–55.8 to 42.1)	**17.5**‡ (5.5 to 29.4)	18.8 (–21.9 to 59.5)
Would recommend this facility	8.1 (–1.9 to 18.2)	2.4 (–4.4 to 9.2)	9.1 (–5.3 to 23.4)
Satisfied with the attitude of the health worker(s)	11.3 (–1.3 to 24.0)	9.0* (0.9 to 18.5)	–1.9 (–20.0 to 16.2)
Satisfied with overall hygiene	–3.3 (–25.4 to 18.9)	22.5 (–5.7 to 50.6)	9.6 (–29.0 to 48.2)
**External nonparticipant observation**			
Effective communication			
HW informs the woman of findings	10.6 (–33.7 to 60.8)	7.8 (–34.9 to 50.7)	–9.7 (–41.8 to 22.4)
HW informs the delivery plan	–33.8* (–62.9 to –4.6)	26.5 (–40.9 to 93.8)	–21.0 (–57.5 to 14.6)
Courteous communication between HW and the woman	16.6 (–49.6 to 82.7)	0.6 (–15.2 to 16.5)	1.8 (–22.2 to 25.9)
Emotional support			
HW is caring and supportive when the woman is in pain	–5.4 (–45.4 to 36.4)	–4.3 (–25.6 to 17.1)	–18.2 (–65.1 to 28.8)
HW refuses a request made (if requested)	–3.6 (–10.3 to 3.1)	3.6 (–6.5 to 13.6)	10.0 (–6.9 to 26.7)
Respectful and dignified care			
Privacy was ensured during the initial examination	8.7 (–28.2 to 56.3)	9.5 (–43.3 to 62.4)	12.4 (–16.2 to 41.0)
Privacy ensured during labour	17.7 (–28.9 to 64.3)	–7.1 (–55.9 to 41.7)	8.2 (–28.9 to 45.3)
Privacy ensured during delivery	–2.8 (–5.0 to 0.5)	–5.3 (–51.6 to 40.9)	2.4 (–19.9 to 24.7)

Difference-in-difference estimates were adjusted by cluster effect at the facility level.

*Denoted<0.05.

†Denoted<0.001.

‡Denoted<0.01.

HW, health worker; RMC, respectful maternity care.

#### Bangladesh

In the intervention group, women reported a statistically significant reduction in physical abuse (DiD −5.2; 95% CI −9.0 to –1.4). In addition, although statistically non-significant, women reported a reduction in verbal abuse (DiD −8.2; 95% CI −16.6 to 0.09). The proportion of women reporting verbal abuse decreased from 8.7% at baseline to 0.9% at endline in the intervention group, while no changes were observed in the control group. Conversely, statistically non-significant improvements were observed in the control group for outcomes that had lower performance at baseline compared with the intervention group. These outcomes were friendly communication (DiD −11.4; 95% CI −48.5 to 26.5) and HW informing the woman of the findings (DiD −9.8; 95% CI −69.9 to 50.1). Other RMC measures showed similar results, as both groups showed enhancements (table 2). Across RMC measures, changes ranged from 8.7% to 22.9% in the intervention group, and 3.5% to 29.8% in the control group. Measures with modest changes typically had very high baseline rates. An exception was the measure ‘satisfaction with general hygiene’, which, despite low satisfaction rates at baseline (10.3% in the intervention group and 8.3% in the control group), had minimal improvements reported (15.3% in the intervention group and 16.6% in the control group).

A statistically significant improvement was shown in observer-reported communication of the delivery plan (DiD −33.8; 95% CI −62.9 to −4.6) in the control group. The proportion of HWs communicating the delivery plan decreased in the intervention group from 86.3% at baseline to 61.8% at endline, while in the control group, the proportion increased from 60.4% to 69.6%. On the other hand, observers reported statistically non-significant improvements in the intervention facilities in these measures: courteous communication between HWs and women (DiD 16.6; 95% CI −49.6 to 82.7), ensuring privacy during labour (DiD 17.7; 95% CI −28.9 to 64.3) and HWs informing findings to women (DiD 10.6; −33.7 to 60.8).

#### Ghana

Women in Ghana reported statistically significant improvements associated with the intervention across various measures of RMC. These improvements included HWs enabling women to ask questions and express concerns (DID 37.5; 95% CI 5.9 to 69.0), considering women’s concerns (DiD 14.9; 95% CI 4.9 to 24.9), being responsive when women asked for support (DiD 5.5; 95% CI 0.7 to 10.3), ensuring privacy (DiD 8.0; 95% CI 0.6 to 16.0), treating women with respect (DiD 5.5; 95% CI 1.0 to 9.9) and reducing verbal (DiD −8.0; 95% CI −12.1 to –3.8) and physical abuse (DiD −5.2; 95% CI −11.4 to –0.9). In addition, more women in the intervention group would select the current facility for another delivery (DiD 17.5; 95% CI 5.5 to 29.4) and they were satisfied with the attitude of health personnel (DiD 9.0; 95% CI 0.9 to 18.5). Women in the intervention group also reported enhanced satisfaction with overall hygiene (DiD 22.5; 95% CI −5.7 to 50.6), although this change was not statistically significant.

Two observer-reported measures showed further but still statistically non-significant improvements associated with the interventions: HWs informing women about the delivery plan (DiD 26.5; 95% CI −40.9 to 93.8) and ensuing privacy during initial examination (DiD 9.5; 95% CI −43.3 to 62.4). Other measures did not show differences, as changes were observed in both groups, including observer-reported privacy, which had notably low rates at baseline.

#### Tanzania

The intervention was associated with statistically significant improvements in women-reported privacy (DiD 24.2; 95% CI 0.2 to 48.3). Women reported other statistically non-significant improvements with the intervention: friendly communication (DiD 20.2; 95% CI −16.3 to 56.6), HW enabling questions and conerns (DiD 14.6; 95% CI −27.1 to 56.4), willingness to return for another delivery (DiD 18.8; 95% CI −21.9 to 59.5) and high satisfaction with care (DiD 17.0; −12.8 to 46.7). Additionally, the proportion of verbal abuse decreased in the intervention group from 7.1% at baseline to 2.3% at endline. In the control group, it decreased from 2.3% to 1.0%. Other measures did not show differences, mainly because rates improved in both groups or were already high at baseline.

No differences between groups were shown in most observer-reported RMC measures due to improvements in both groups or maintenance of high baseline rates. A statistically non-significant improvement was seen in observer-report privacy during the initial examination (DiD 12.4; −16.2 to 41.0) in the intervention group. Conversely, in the control group, positive trends were noted in observer assessments of HW informing women about the delivery plan (DiD -21.0 to −57.5,14.6) and providing support when women were in pain (DiD −18.2; −65.1 to 28.8).

### Environmental factors potentially enabling or acting as barriers to RMC

In Bangladesh, similar trends with input measures were observed in both groups. The labour ward layout improved in both groups. Women’s satisfaction with labour ward toilet cleanliness showed minimal change, despite very low baseline rates (table 4). On the other hand, substantial and statistically significant improvements were reported by HWs in RMC training (DiD 59.0; 95% CI 33.4 to 84.6) and the availability of policy/procedures for both addressing patients’ concerns (DiD 46.0; 95% CI 4.7 to 87.4) and identifying/reporting abuse (DiD 45.9; 95% CI 19.9 to 71.8) (table 4).

**Table 4:** Environmental factors potentially enabling or acting as barriers to RMC by group, time of evaluation and country

Country	Input measures	Pre-intervention		Post-intervention		Difference-in-difference* (%; 95% CI)
		Intervention	Control	Intervention	Control	
		%	%	%	%	
Bangladesh	Physical and human resources (observations)	**N=167**	**N=221**	**N=189**	**N=271**	
	Labour ward is open (no individual rooms or curtains)	20.7	41.4	6.6	21.7	–5.6 (–72.4 to 65.7)
	Occupancy: all beds were occupied	26.8	26.4	38.6	35.5	–2.7 (–43.2 to 49.3)
	Number of health workers on duty (mean; SD)	4.1±0.86	2.3±0.08	4.0±3.56	3.1±0.22	–0.9 (–2.0 to 24.1)
	Labour toilet was very clean†	11.1	6.6	18.9	9.7	4.6 (–9.2 to 18.4)
	Policies, procedures, and training (health workers’ interviews)	**N=53**	**N=105**	**N=92**	**N=166**	
	There are policies/procedures for addressing patient concerns	38.5	43.7	84.8	44.0	**46.0**‡ (4.7 to 87.4)
	Have clear policies on patient’s rights	67.3	46.6	94.6	61.5	12.4 (–30.7 to 55.6)
	There is a process for identifying and reporting abuse	28.3	29.5	82.6	38.0	**45.9§** (19.9 to 71.8)
	Received training on RMC	39.6	41.0	79.4	21.7	**59.0**§ (33.4 to 84.6)
Ghana	Physical and human resources (observations)	**N=76**	**N=57**	**N=154**	**N=129**	
	Labour ward is open (no individual rooms or curtains)	96.1	84.2	31.4	75.6	–56.0 (–112.5 to 0.4)
	Occupancy: all beds were occupied	52.6	54.4	73.0	42.2	**32.6‡** (9.9 to 64.3)
	Number of health workers on duty (mean; SD)	3.1±0.13	3.4±0.23	4.7±0.2	3.7±0.2	**1.39**¶ (0.3 to 3.3)
	Labour toilet was very clean†	30.4	51.0	56.8	64.1	13.4 (–32.0 to 58.8)
	Policies, procedures and training (health workers’ interviews)	**N=44**	**N=42**	**N=63**	**N=59**	
	There are policies/procedures for addressing patient concerns	75.6	64.3	90.5	59.6	19.6 (–7.6 to 46.7)
	Have clear policies on patient’s rights	73.3	90.5	96.9	94.8	19.2 (–4.5 to 42.8)
	There is a process for identifying and reporting abuse	73.3	61.9	77.1	75.9	–8.9 (–46.3 to 28.4)
	Received training on RMC	66.7	73.8	73.4	81.0	–0.5 (–27.3 to 23.4)
Tanzania	Physical and human resources (clinical observations)	**N=65**	**N=55**	**N=68**	**N=59**	
	Labour ward is open (no individual rooms or curtains)	10.8	14.6	0.0	0.0	3.8 (–28.3 to 35.8)
	Occupancy: all beds were occupied	4.6	41.8	7.4	22.6	22.0 (–46.4 to 90.4)
	Number of health workers on duty (mean; SD)	2.6±0.15	2.3±0.17	2.4±0.19	2.9±0.21	–0.7 (–1.7 to 3.3)
	Labour toilet was clean†	0.0	37.9	32.4	49.3	21.0 (–18.1 to 60.0)
	Policies, procedures and training (health workers’ interviews)	**N=41**	**N=40**	**N=55**	**N=58**	
	There are policies/procedures for addressing patient concerns	56.1	57.5	81.8	91.4	–8.2 (–58.2 to 41.9)
	Have clear policies on patient’s rights	70.7	70.0	83.6	96.6	–13.6 (–60.4 to 33.1)
	There is a process for identifying and reporting abuse	34.2	62.5	54.6	60.3	22.6 (–20.0 to 65.1)
	Received training on RMC	34.2	47.5	60.0	68.9	4.4 (–31.7 to 40.5)

Denominators vary for each measure because the same sections were not observed in all women, as some women were admitted due to complications, needed caesarean section, or were admitted in the second stage of labour.

*Adjusted by clustering effect at the facility level.

†Assessed among women reported having used the toilet during labour.

‡Denoted<0.05.

§Denoted<0.001.

¶Denoted <0.01.

RMC, respectful maternity care.

In Ghana, there was a statistically significant increase in the mean number of staff on duty—mostly midwives (DiD 1.39; 95% CI 0.3 to 3.3)—in the intervention group, and a reduction of open-layout labour wards (DiD −56.0; 95% CI −112.5 to 0.4). At the same time, there was an increase in the intervention group in the proportion of observations, in which all labour ward beds were occupied (DiD 32.6; 95% CI 9.9 to 64.3). In addition, HWs were more likely to report improvements in the availability of both policies/procedures for addressing patient concerns (DiD 19.6; 95% CI −7.6 to 46.7) and clear policies on patients’ rights (DiD 19.2; 95% CI −4.5 to 42.8). There was no difference between groups in the availability of procedures for identifying and reporting abuse or training in RMC.

In Tanzania, the results suggest a favourable trend in the cleanliness of labour toilets in intervention facilities (DiD 21.0; 95% CI −18.1 to 60.0) and an increase in the occupancy of labour wards (DiD 22.0; 95% CI −46.4 to 90.4). No changes were observed in labour ward layouts and staff availability. Although statistically non-significant, an improvement was reported in intervention facilities in the availability of a process for identifying and reporting abuse (DiD 22.6; 95% CI −20.0 to 65.1). Conversely, greater improvements were reported in control facilities in the availability of clear policies on patient rights (DiD −13.6; −60.4 to 3.1).

Finally, we measured the impact of introducing the QoC standards on HWs’ self-assessed provision of RMC (online supplemental table S3). Tanzania was the only site where HWs in intervention facilities reported a statistically significant improvement over time on self-assessed provision of RMC (DiD 0.84; 95% CI 0.21,1.47).

## Discussion

Implementing MNH quality standards for 18 months under real-world health system conditions was associated with some improvements in RMC measures. There was a larger trend in reducing physical and verbal abuse, enhancing privacy and increasing women’s satisfaction in the intervention facilities. However, other measures, such as effective communication and emotional support, showed no difference or varied substantially across countries. In most cases, where no differences were detected, both groups either improved or maintained high rates throughout the study. The availability of policies/procedures addressing patient concerns, patient rights and identifying/reporting abuse increased more in intervention facilities than in control facilities. Nevertheless, the results related to cleanliness, the availability of human resources and their training varied substantially across countries. Only in Ghana did the implementation of the MNH standards show consistent improvements over time across most domains and measures. Overall, women more often reported improvements in RMC than external observers.

Most of the body of evidence has focused on measuring RMC adoption gaps and validating RMC measurement methods.^9^ Non-comparative studies consistently suggest improvements in RMC with implemented interventions.^15 31–33^ However, we identified that RMC measures in intervention and control facilities tend to improve. This may be because the need for RMC is already clearly identified and puts pressure on the entire health system towards improvement or due to contamination, given the nature of the multilevel intervention.^34 35^ Either way, uncontrolled pre–post intervention studies fail to identify whether the improvements are associated with the intervention or secular trends. Very few comparative studies measured the effect of interventions to improve RMC and showed divergent results.^11 17 36^ Some authors attribute this to the multiple and complex challenges of implementing change in a low-resource setting and variations in measurement.^9 17^ The study that obtained similar results to ours is the one that implemented a complex strategy, which was designed in a participatory manner with multiple stakeholders and levels of leadership, specific to the context and supported structural improvements.^11^ Contrary to the most commonly used implementation strategy (the training of HWs), complex, multilevel, context-specific implementation strategies addressing a broad spectrum of barriers (including contextual factors) may be more effective in accelerating the adoption of RMC.^16 37^

Consistent with the literature, women reported more significant improvements in care experience compared with observers.^38 39^ This may suggest that women may have lower expectations of care experiences than observers or may indicate a possible social desirability bias. These findings further highlight the importance of the discussions around which source to use in measuring RMC objectively, considering potential biases and the cost of the different data collection approaches.

Some results are of particular interest. Intervention facilities in Ghana showed improvements of greater magnitude compared with other countries. The literature reports higher adoption of RMC in midwifery-led care services and lower workload.^40 41^ Given that the facilities in Ghana had the highest proportion of midwives and young personnel and that facilities in the intervention group significantly increased the available staff, the question arises as to whether the intervention could have better penetration among young midwives or new personnel. There remains a critical gap in women’s satisfaction with hygiene in Bangladesh and Tanzania. Knowing that women reported high satisfaction levels for other dimensions, these proportions may indicate that facility hygiene is a priority for women and was not addressed in some countries. Finally, emotional support did not improve because the adoption was already high at baseline in Tanzania, but other factors could have played a role in Bangladesh.

This study had several key strengths. It was a prospective multicountry comparative study conducted in 43 health facilities in three countries across two world regions. The intervention was implemented at a large scale (multiple districts) with the participation of numerous key stakeholders and substantial local input and leadership. The data collection involved mixed methods integrating the views of women, HWs and independent observers. It was a study conducted in real-life routine practice conditions, including diverse healthcare facilities, facilitating the generalisability or applicability of the findings to many similar settings around the world.^42^ It implemented the standards of care as a package using various standardised methods described in globally available guidelines that could facilitate their reproducibility. The inclusion of trained independent external observers was another strength.

Our study had some limitations. While a controlled before and after analysis is more robust than non-comparative studies, it does have limitations in controlling potential confounding.^43^ These effects were minimised by estimating DiD, which assumes that baseline rates are different and compares measure changes over time between groups to obtain an appropriate counterfactual for estimating a causal effect. Nevertheless, since the outcomes of interest are not typically collected in routine health information systems and collecting baseline primary data at multiple points in time was not feasible, we were unable to determine differences in pretreatment trends. Nonetheless, our analysis compared facilities and participants with many similar characteristics, for which parallel trends seem plausible. A ceiling effect was observed for some measures, benefiting the group with lower rates. Second, the country-specific number of clusters may not have been enough to detect some clinically significant point estimates as statistically significant. In addition, a few large facilities may have contributed most of the samples and had most of the improvements. Third, some high RMC rates could have been biased by the Hawthorne effect, which may have overestimated frequencies. We mitigated this with 2 weeks of continuous observation, which we considered sufficient time for participants to return to normal behaviours.

## Conclusion

This study provides evidence that implementing MNH QoC standards could accelerate the improvement of some RMC measures in LMICs. Participatory designs are likely to encourage engagement, ownership, and capacity share at multiple levels, potentially driving systems strengthening to achieving universal health coverage with quality and respectful care. Using context-specific solutions may contribute to advancing RMC, provided there is adequate investment and support. The results suggest that a scale-up of implementing MNH QoC standards in LMICs and accelerating women’s access to RMC is feasible and must be a desired goal.

## Data Availability

Data are available upon reasonable request.

## References

[1] Oladapo OT, Tunçalp Ö, Bonet M, et al. WHO model of Intrapartum care for a positive childbirth experience: transforming care of women and babies for improved health and wellbeing. BJOG 2018;125:918–22. 10.1111/1471-0528.15237 29637727 PMC6033015

[2] WHO recommendation on respectful maternity care during labour and childbirth [WHO - RHL]. 2023 Available: https://srhr.org/rhl/article/who-recommendation-on-respectful-maternity-care-during-labour-and-childbirth

[3] Bohren MA, Vogel JP, Hunter EC, et al. The mistreatment of women during childbirth in health facilities globally: a mixed-methods systematic review. PLoS Med 2015;12:e1001847. 10.1371/journal.pmed.1001847 26126110 PMC4488322

[4] Bohren MA, Hunter EC, Munthe-Kaas HM, et al. Facilitators and barriers to facility-based delivery in low- and middle-income countries: a qualitative evidence synthesis. Reprod Health 2014;11. 10.1186/1742-4755-11-71 PMC4247708 25238684

[5] Moyer CA, Adongo PB, Aborigo RA, et al. 'They treat you like you are not a human being': maltreatment during labour and delivery in rural northern Ghana. Midwifery 2014;30:262–8. 10.1016/j.midw.2013.05.006 23790959

[6] Chadwick RJ, Cooper D, Harries J. Narratives of distress about birth in South African public maternity settings: a qualitative study. Midwifery 2014;30:862–8. 10.1016/j.midw.2013.12.014 24456659

[7] Statement WHO. The prevention and elimination of disrespect and abuse during facility-based childbirth. Available: https://apps.who.int/iris/bitstream/handle/10665/134588/WHO_RHR_14.23_eng.pdf [Accessed 23 Mar 2023].

[8] Shakibazadeh E, Namadian M, Bohren MA, et al. Respectful care during childbirth in health facilities globally: a qualitative evidence synthesis. BJOG 2018;125:932–42. 10.1111/1471-0528.15015 29117644 PMC6033006

[9] Diamond-Smith N, Lin S, Peca E, et al. A landscaping review of interventions to promote respectful maternal care in Africa: opportunities to advance innovation and accountability. Midwifery 2022;115. 10.1016/j.midw.2022.103488 36191382

[10] Sen G, Reddy B, Iyer A. Beyond measurement: the drivers of disrespect and abuse in obstetric care. Reprod Health Matters 2018;26:6–18. 10.1080/09688080.2018.1508173 30189791

[11] Kujawski SA, Freedman LP, Ramsey K, et al. Community and health system intervention to reduce disrespect and abuse during childbirth in Tanga region, Tanzania: a comparative before-and-after study. PLoS Med 2017;14:e1002341. 10.1371/journal.pmed.1002341 28700587 PMC5507413

[12] Warren CE, Njue R, Ndwiga C, et al. Manifestations and drivers of mistreatment of women during childbirth in Kenya: implications for measurement and developing interventions. BMC Pregnancy Childbirth 2017;17. 10.1186/s12884-017-1288-6 PMC5371243 28351350

[13] Ameh CA, White S, Dickinson F, et al. Retention of knowledge and skills after emergency obstetric care training: a multicountry longitudinal study. PLoS One 2018;13:e0203606. 10.1371/journal.pone.0203606 30286129 PMC6171823

[14] Asefa A, Morgan A, Bohren MA, et al. Lessons learned through respectful maternity care training and its implementation in Ethiopia: an Interventional mixed methods study. Reprod Health 2020;17. 10.1186/s12978-020-00953-4 PMC7331171 32615999

[15] Abuya T, Ndwiga C, Ritter J, et al. The effect of a multi-component intervention on disrespect and abuse during childbirth in Kenya. BMC Pregnancy Childbirth 2015;15:224. 10.1186/s12884-015-0645-6 26394616 PMC4580125

[16] Downe S, Lawrie TA, Finlayson K, et al. Effectiveness of respectful care policies for women using routine Intrapartum services: a systematic review. Reprod Health 2018;15:23. 10.1186/s12978-018-0466-y 29409519 PMC5801845

[17] Sudhinaraset M, Giessler KM, Nakphong MK, et al. Can a quality improvement intervention improve person-centred maternity care in Kenya? Sex Reprod Health Matters 2023;31:2175448. 10.1080/26410397.2023.2175448 36857118 PMC9980034

[18] Afulani PA, Aborigo RA, Walker D, et al. Can an integrated obstetric emergency simulation training improve respectful maternity care? Results from a pilot study in Ghana. Birth 2019;46:523–32. 10.1111/birt.12418 30680785

[19] Tunçalp Ӧ, Were WM, MacLennan C, et al. Quality of care for pregnant women and newborns-the WHO vision [122]. BJOG 2015;122:1045–9. 10.1111/1471-0528.13451 25929823 PMC5029576

[20] Lazzerini M, Valente EP, Covi B, et al. Use of WHO standards to improve quality of maternal and newborn hospital care: a study collecting both mothers' and staff perspective in a tertiary care hospital in Italy. BMJ Open Qual 2019;8:e000525. 10.1136/bmjoq-2018-000525 PMC6440608 30997420

[21] Ogrinc G, Davies L, Goodman D, et al. SQUIRE 2.0 (standards for quality improvement reporting excellence): revised publication guidelines from a detailed consensus process. BMJ Qual Saf 2016;25:986–92. 10.1136/bmjqs-2015-004411 PMC5256233 26369893

[22] Statistics and Informatics Division Ministry of Planning. Bangladesh - population and housing census 2011. Government of Bangladesh; 2011.

[23] Ghana Health Service (GHS), ICF International. Ghana demographic and health survey. Ghana Statistical Service (GSS); 2014.

[24] United Republic of Tanzania 2012 Population and Housing Census. Dar es Salaam: National Bureau of Statistics, Ministry of finance; 2013.

[25] Reinertsen JL, Bisognano M, Pugh MD. Seven leadership leverage points for Organisation-level improvement in health care. In: IHI Innovation Series white paper. Cambridge, MA: Institute for Healthcare Improvement, 2008.

[26] Reinertsen JL, Gosfield AG. Engaging Physicians in a Shared Quality Agenda. Cambridge, MA, 2007.

[27] Silimperi DR, Franco LM, Zanten T, et al. A framework for Institutionalising quality assurance. Int J 2002;14:67–73. 10.1093/intqhc/14.suppl_1.67 12572789

[28] World Health Organisation. A network for improving quality of care for maternal, newborn and child health [World Health Organisation]. 2019. Available: https://cdn.who.int/media/docs/default-source/mca-documents/qoc/qed-quality-of-care-for-maternal-and-newborn-health-a-monitoring-framework-for-network-countries.pdf?sfvrsn=19a9f7d0_1&download=true

[29] World Health Organisation. Standards for improving quality of maternal and newborn care in health facilities. 2016. Available: https://cdn.who.int/media/docs/default-source/mca-documents/qoc/quality-of-care/standards-for-improving-quality-of-maternal-and-newborn-care-in-health-facilities.pdf

[30] Respectful maternity care delivered within health facilities in Bangladesh, Ghana and Tanzania: a cross- sectional assessment preceding a quality improvement intervention; 10.1136/bmjopen-2020-039616 PMC7818820 33472772

[31] Kongnyuy EJ, Mlava G, van den Broek N. Criteria-based audit to improve women-friendly care in maternity units in Malawi. J Obstet Gynaecol Res 2009;35:483–9. 10.1111/j.1447-0756.2008.00990.x 19527387

[32] Mihret H, Atnafu A, Gebremedhin T, et al. Reducing disrespect and abuse of women during antenatal care and delivery services at Injibara general hospital, Northwest Ethiopia: a prepost Interventional study. Int J Womens Health 2020;12:835–47. 10.2147/IJWH.S273468 33116933 PMC7568622

[33] Zethof S, Bakker W, Nansongole F, et al. Prepost implementation survey of a multicomponent intervention to improve informed consent for caesarean section in Southern Malawi. BMJ Open 2020;10:e030665. 10.1136/bmjopen-2019-030665 PMC6955547 31911511

[34] Chen Y-F, Hemming K, Stevens AJ, et al. Secular trends and evaluation of complex interventions: the rising tide phenomenon. BMJ Qual Saf 2016;25:303–10. 10.1136/bmjqs-2015-004372 PMC4853562 26442789

[35] Manu A, Billah SM, Williams J, et al. Institutionalising maternal and newborn quality-of-care standards in Bangladesh, Ghana and Tanzania: a quasi-experimental study. BMJ Glob Health 2022;7:e009471. 10.1136/bmjgh-2022-009471 PMC9490604 36130773

[36] Brown H, Hofmeyr GJ, Nikodem VC, et al. Promoting childbirth companions in South Africa: a randomised pilot study. BMC Med 2007;5:7. 10.1186/1741-7015-5-7 17470267 PMC1905915

[37] Althabe F, Chomba E, Tshefu AK, et al. A multifaceted intervention to improve Syphilis screening and treatment in pregnant women in Kinshasa, democratic Republic of the Congo and in Lusaka, Zambia: a cluster randomised controlled trial. Lancet Glob Health 2019;7:e655–63. 10.1016/S2214-109X(19)30075-0 30910531 PMC6465956

[38] Esan OT, Maswime S, Blaauw D. Directly observed and reported respectful maternity care received during childbirth in public health facilities, Ibadan metropolis, Nigeria. PLoS ONE 2022;17:e0276346. 10.1371/journal.pone.0276346 36269737 PMC9586397

[39] Freedman LP, Kujawski SA, Mbuyita S, et al. Eye of the beholder? Observation versus self-report in the measurement of disrespect and abuse during facility-based childbirth. Reprod Health Matters 2018;26:107–22. 10.1080/09688080.2018.1502024 30199353

[40] Sandall J, Soltani H, Gates S, et al. Midwife-led continuity models versus other models of care for childbearing women. Cochrane Database Syst Rev 2013:CD004667. 10.1002/14651858.CD004667.pub3 23963739

[41] Dynes MM, Twentyman E, Kelly L, et al. Patient and provider determinants for receipt of three dimensions of respectful maternity care in Kigoma region, Tanzania-April-July, 2016. Reprod Health 2018;15:41. 10.1186/s12978-018-0486-7 29506559 PMC5838967

[42] Patsopoulos NA. A pragmatic view on pragmatic trials. Dialogues Clin Neurosci 2011;13:217–24. 10.31887/DCNS.2011.13.2/npatsopoulos Available: 10.31887/DCNS.2011.13.2/npatsopoulos 21842619 PMC3181997

[43] Coly A, Parry G. Evaluating Complex Health Interventions: A Guide to Rigorous Research Designs. AcademyHealth, 2017.

