# Supplementary material for: Implementing maternal and newborn health quality of care standards in healthcare facilities to improve the adoption of respectful maternity care in Bangladesh, Ghana and Tanzania: a controlled before and after study
Source: BMJ Glob Health. 2023 Nov 14;8(11):e012673. doi: 10.1136/bmjgh-2023-012673 (PMC10649771; doi:10.1136/bmjgh-2023-012673)
Supplement: Supplementary data [file bmjgh-2023-012673supp001.pdf]

Supplementary materials

Supplementary Tables

Supplementary Table S1. Aggregated characteristics of participating healthcare facilities

Supplementary Table S2. Characteristics of each participating healthcare facility

Supplementary Table S3. Effect of introducing quality of care standards on labour companionship and provider self-reported provision of RMC

Supplementary Table S4: Baseline and endline characteristics of participating women, health workers, and observations by group and country

Supplementary File

Supplementary SF1. Reflexivity Statement

Supplementary Tables

Supplementary Table S1. Characteristics of participating health care facilities

	Bangladesh				Ghana				Tanzania			
	Intervention		Control		Intervention		Control		Intervention		Control	
Variables	**Baseline N=5**	**Endline N=5**	**Baseline N=10**	**Endline N=10**	**Baseline N=8**	**Endline N=8**	**Baseline N=8**	**Endline N=8**	**Baseline N=6**	**Endline N=6**	**Baseline N=6**	**Endline N=6**
Type of facility												
Health centre	4	4	8	8	3	3	3	3	4	4	4	4
Hospital	1	1	2	2	5	5	5	5	2	2	2	2
Facility ownership												
Government	5	5	10	10	6	6	7	7	4	4	3	3
Private/FBO/NGO	0	0	0	0	2	2	1	1	2	2	3	3
Facility Designation												
BEmONC	4	1	2	1	4	5	5	5	2	2	2	2
CEmONC	1	3	8	9	4	3	3	3	4	4	4	4
Missing	0	1	0	0	0	0	0	0	0	0	0	0
Number of beds												
<50	1	0	6	1	5	5	5	6	5	4	2	3
50-100	3	4	4	8	1	1	0	0	0	0	2	1
>100	1	1	0	1	2	1	3	0	1	2	2	2
Missing	0	0	0	0	0	1	0	2	0	0	0	0
The facility admits patients overnight												
Yes	5	5	10	10	7	7	7	7	6	6	6	6
No	0	0	0	0	1	1	1	1	0	0	0	0

Supplementary Table S2. Characteristics of each participating healthcare facility

Facility code	Number of beds	Governance	Level of care	Total births in 2016 (singletons and multiples)	Maternity care model
Bangladesh					
Intervention					
A	250	Public	Tertiary hospital	1126	Obstetrician-led
B	50	Public	Primary Health Care Center	773	Shared
C	31	Public	Primary Health Care Center	555	Shared
D	50	Public	Primary Health Care Center	67	Shared
E	50	Public	Primary Health Care Center	50	Shared
Control					
F	100	Public	Secondary hospital	830	Obstetrician-led
G	100	Public	Secondary hospital	414	Obstetrician-led
H	50	Public	Primary Health Care Center	360	Shared
I	50	Public	Primary Health Care Center	368	Shared
J	31	Public	Primary Health Care Center	363	Shared
K	31	Public	Primary Health Care Center	302	Shared
L	31	Public	Primary Health Care Center	110	Shared
M	31	Public	Primary Health Care Center	253	Shared
N	31	Public	Primary Health Care Center	212	Shared
O	31	Public	Primary Health Care Center	186	Shared
P	343	Public-NGO/FBO	District Hospital	5016	Shared Model of Care
Ghana					
Intervention					
A	226	Public	Regional Hospital	3789	Shared Model of Care
B	70	Public	District Hospital	1207	Shared Model of Care
C	17	Public	Primary Health Care Center	456	Midwife-led care
D	10	Public	Primary Health Care Center	288	Midwife-led care

Facility code	Number of beds	Governance	Level of care	Total births in 2016 (singletons and multiples)	Maternity care model
E	8	Public	Primary Health Care Center	277	Midwife-led care
F	7	Public	Primary Health Care Center	269	Midwife-led care
G	6	Public-NGO/FBO	Primary Health Care Center	131	Midwife-led care
Control					
H	164	Public	District Hospital	1561	Shared Model of Care
I	119	Public	District Hospital	867	Shared Model of Care
J	110	Public	District Hospital	1728	Shared Model of Care
K	28	Public	Primary Health Care Center	443	Midwife-led care
L	25	Public	Primary Health Care Center	523	Midwife-led care
M	17	Public-NGO/FBO	Primary Health Care Center	303	Midwife-led care
N	9	Public	Primary Health Care Center	86	Midwife-led care
O	5	Public	Primary Health Care Center	137	Midwife-led care
Tanzania					
Intervention					
A	150	Public	District Hospital	1482	Shared
B	37	NGO/FBO	FBO Hospital	1566	Shared
C	30	Public	Primary Health Care Center	95	Shared
D	28	NGO/FBO	Primary Health Care Center	126	Shared
E	24	Public	Primary Health Care Center	52	Shared
F	13	Public	Primary Health Care Center	52	Shared
Control					
G	450	NGO/FBO	FBO Hospital	1600	Shared
H	130	Public	District Hospital	658	Shared
I	80	NGO/FBO	Primary Health Care Center	320	Shared
J	50	Private	Primary Health Care Center	157	Shared
K	26	Public	Primary Health Care Center	227	Shared

Facility code	Number of beds	Governance	Level of care	Total births in 2016 (singletons and multiples)	Maternity care model
L	26	Public	Primary Health Care Center	232	Shared

NGO: Non-government organization; FBO: Faith-based organization; Midwife-led care model was selected to describe settings where the midwife plans, organize and delivers care to healthy women with uncomplicated or 'low-risk' pregnancies. Some antenatal and/or intrapartum and/or postpartum care may be provided in consultation with medical staff as appropriate. Within these models, midwives are, however, in partnership with the woman, the lead professional with responsibility for assessment of her needs, planning her care, referral to other professionals as appropriate, and for ensuring provision of maternity services. Obstetrician-provided care model was selected where the primary providers of care for most childbearing women (including low-risk) is an obstetrician. An obstetrician (not necessarily the one who provides care) is present for the birth, and nurses follow obstetrician's directions. Shared models of care involve shared responsibility for the organisation and delivery of care, throughout initial booking to the postnatal period, is shared between different health professionals.

Supplementary Table S3. Effect of introducing quality of care standards on labour companionship and provider self-reported provision of RMC

	Pre-intervention		Post-intervention		Difference-in-Difference adjusted[1] (95% CI)
RMC practices	Intervention %	Control %	Intervention %	Control %	
**Bangladesh**	**N=53**	**N=105**	**N=92**	**N=166**	
**Heath workers interviews**					
HW self-reported provision of RMC (means; SD)	4.1 (0.16)	4.2 (0.10)	4.0 (0.61)	3.8 (0.07)	-0.27 (-0.90;3.7)
**Ghana**	**N=44**	**N=42**	**N=63**	**N=59**	
**Heath workers interviews**					
HW self-reported provision of RMC (means; SD)	4.2 (0.53)	4.1 (0.5)	4.2 (0.9)	4 (0.8)	0.1 (-0.3;0.5)
**Tanzania**	**N=41**	**N=40**	**N=55**	**N=58**	
**Heath workers interviews**					
HW self-reported provision of RMC (means; SD)	3.4 (0.13)	4.0 (0.1)	4.2 (0.12)	3.9 (0.09)	**0.84*** (0.21;1.47)

Note: HW: health worker. [1]Adjusted by clustering effect at the facility level. *Denoted <0.05; ** Denoted <0.01; ***Denoted <0.001.

Supplementary Table S4. Baseline and endline characteristics of participating women, health workers, and observations by group and country

Variables	Bangladesh				Ghana				Tanzania			
	Baseline		Endline		Baseline		Endline		Baseline		Endline	
	Intervention	Control	Intervention	Control	Intervention	Control	Intervention	Control	Intervention	Control	Intervention	Control
	%	%	%	%	%	%	%	%	%	%	%	%
Interviewed women	N=126	N=169	N=111	N=157	N=210	N=171	N=291	N=232	N=86	N=88	N=86	N=100
Age in years												
Less than 20	32.5	17.8	27.7	23.1	14.3	12.3	15.4	16.5	17.6	17.1	15.1	18.0
20-34	60.3	76.3	66.1	69.9	71.9	72.5	71.6	70.6	69.4	73.9	69.8	78.0
35 o more	7.1	5.9	6.3	7.1	13.8	15.2	13.0	13.0	12.9	9.1	15.1	4.0
Education level												
Primary incomplete/no schooling	31.0	30.8	29.7	27.5	34.3	32.8	28.5	27.7	5.9	12.5	11.6	13.0
Primary complete	44.4	36.6	40.5	36.9	25.2	25.2	18.2	19.1	68.2	69.3	68.6	52.0
Middle/Secondary	24.6	32.5	29.7	35.6	35.2	38.6	41.2	43.3	18.8	17.1	17.4	33.0
Post-secondary education	NA	NA	NA	NA	5.2	3.5	12.0	10.0	7.1	1.1	2.3	2.0
Number of deliveries (including this one)												
1	50.8	53.9	56.3	51.3	32.4	30.4	35.3	33.8	42.6	39.8	33.7	39.0
2-3	42.1	39.1	40.2	41.0	47.6	42.7	39.4	45.0	36.5	42.1	43.0	48.0
4 or more	7.1	7.1	3.6	7.7	20.0	26.9	25.3	21.2	21.2	18.2	23.3	13.0
Interviewed health workers	N=53	N=105	N=92	N=166	N=44	N=42	N=63	N=59	N=41	N=40	N=55	N=58
Age in years												
<30	20.8	16.2	25.0	20.5	40.9	45.2	41.3	42.4	26.8	45.0	29.1	39.7
30 and <40	34.0	47.6	42.4	50.0	29.5	26.2	39.7	34.2	36.6	27.5	36.4	32.8
>=40	45.3	36.2	32.6	29.5	29.5	28.6	19.0	23.4	36.6	27.5	34.6	27.6
Professional role												
Nurse	47.2	63.8	62.0	62.1	18.2	14.3	25.8	17.7	29.3	25.0	29.1	17.2
Midwife	3.8	1.9	10.9	19.9	65.9	73.8	56.5	63.2	43.9	30.0	47.3	46.6
Doctor	32.1	26.7	17.4	16.3	4.5	7.1	0	3.5	17.1	27.5	5.6	12.1

Manu A, *et al. BMJ Glob Health* 2023; 8:e012673. doi: 10.1136/bmjgh-2023-012673

Variables	Bangladesh				Ghana				Tanzania			
	Baseline		Endline		Baseline		Endline		Baseline		Endline	
	Intervention	Control	Intervention	Control	Intervention	Control	Intervention	Control	Intervention	Control	Intervention	Control
	%	%	%	%	%	%	%	%	%	%	%	%
Other	17.0	7.6	9.8	1.8	11.4	4.8	17.7	14.0	9.8	17.5	18.2	24.1
Number of years in current position												
<=5	67.9	57.2	73.9	77.7	25.0	16.7	14.3	22.0	87.8	75.0	61.8	72.4
>5 -10	5.7	9.5	12.0	7.8	9.1	16.7	15.9	10.2	4.9	10.0	18.2	15.5
>10	26.4	33.3	14.2	14.5	65.9	66.7	69.8	67.8	7.3	15.0	20.0	12.1
Gender												
Female	66.0	77.1	83.7	87.4	88.6	81.0	80.6	80.7	78.1	65.0	76.4	70.7
Male	34.0	22.9	16.3	12.7	11.4	19.0	19.4	22.8	21.9	35.0	23.6	29.3
Observations during labour and childbirth	N=167	N=221	N=189	N=271	N=76	N=58	N=154	N=129	N=65	N=55	N=68	N=59
Level of healthcare facility												
Health centre	53.9	58.6	62.0	67.9	14.5	17.2	24.2	23.7	27.7	41.8	30.9	40.7
Hospital	46.1	41.4	38.0	32.1	85.5	82.8	75.8	76.3	72.3	58.2	69.1	59.3
Day of the week												
Monday to Friday	74.7	71.0	85.4	86.4	65.8	75.9	78.7	80.0	67.7	72.7	75.0	81.4
Saturday or Sunday	25.3	29.0	14.6	13.7	34.2	25.1	21.3	20.0	32.3	27.3	25.0	18.6
Moment of arrival												
Day	66.1	60.5	71.35	63.1	47.4	46.5	42.2	54.3	64.6	50.9	64.7	55.9
Night	33.6	37.4	28.6	36.9	43.4	41.4	47.8	45.7	35.4	49.1	35.3	44.1
Missing	0	0	0	0	9.2	12.1	24.2	0	0	0	0	0
Maternal age in years												
Less than 20	18.6	17.6	16.1	20.3	9.2	10.3	14.0	20.6	18.5	12.7	10.3	13.6
20-34	77.3	76.6	76.6	73.4	63.2	56.9	51.0	61.0	67.7	72.7	75.0	78.0
35 o more	4.2	5	7.3	6.27	15.8	25.9	20.4	11.5	0	0	0	0

Note: Bangladesh did not measure post-secondary education as a separate category; the highest education level category was intermediate/secondary or higher.

Supplementary SF1. Reflexivity Statement

1. **How does this study address local research and policy priorities?**

Our study was designed to evaluate the impact of using the maternal and child health quality of care standards to locally develop an implementation strategy to improve measures of Respectful Maternity Care. This issue is of significant priority worldwide, most notably in low- and middle-income countries where health professionals often work in overburdened and under-resourced environments. This study was designed in collaboration with local researchers and agreement and with substantial input from national and sub-national local authorities from Bangladesh, Ghana and Tanzania.

2. **How were local researchers involved in study design?**

This study was designed collaboratively by researchers from Bangladesh, Ghana and Tanzania, with overall coordination by AM based in Ghana and TH based the US. The researchers were involved in the study's design, conduct, monitoring, data management, analysis, and interpretation. Most authors, including the first author, are based in LMIC, and only three out of sixteen (TH, IJ, and VP) are in HIC.

3. **How has funding been used to support the local research team?**

UNICEF funded this study and covered all the costs of conducting the study, as well as investments in the infrastructure and equipment of the hospitals to create enabling environments to practice change.

4. **How are the research staff who conducted data collection acknowledged?**

The teams responsible for conducting the study in each country are co-authors of this publication.

5. **Do all members of the research partnership have access to study data?**

All members of the partnership have access to data.

6. **How was data used to develop analytical skills within the partnership?**

All three countries have data centres and researchers trained in data management and analysis.

7. How have research partners collaborated in interpreting study data?

The first draft of the manuscript was circulated to all co-authors who provided their input. A new edited version was generated, incorporating all the co-authors' suggestions. Finally, all co-authors approved the submission.

8. How were research partners supported to develop writing skills?

Veronica Pingray (doctoral candidate in Epidemiology) drafted the manuscript with the supervision and guidance of two senior researchers (AM and TH) and the input and approval of all co-authors.

9. How will research products be shared to address local needs?

This study will be published in a journal as open access. UNICEF has developed a post-publication dissemination plan. This will include the dissemination among leaders in global health, including researchers, programme officers, policymakers and implementers based in LMICs.

10. How is the leadership, contribution and ownership of this work by LMIC researchers recognised within the authorship?

AM is the first author since he coordinated the research and for that reason, he is the first author. VP corrected the databases, analysed the data and drafted the manuscript. She is a mid-level career researcher pursuing her PhD in perinatal epidemiology. Her work is acknowledged as a joint first author. All other authors reviewed and approved the manuscript and are co-authors. The authorship team is predominantly based in LMICs. TH is the senior co-author (based in HIC) given her seniority level and because she supervised, advised and made decisions during the study and the development of the manuscript.

11. **How have early career researchers across the partnership been included within the authorship team?**

We have included early career researchers (VP and IJ) among the co-authors. They worked hard under the supervision of senior authors, and VP is the first joint co-author.

12. How has gender balance been addressed within the authorship?

Seven authors are male (AM, SKM, JW, FY, PM, SE, MZM and DM), and nine authors are female (VP, TH, SK, FG, PW, FK, MV, NB, IJ)

13. How has the project contributed to the training of LMIC researchers?

All authors in low- and middle-income countries are senior researchers. Research funding provided financial support to the three sites to set up and hire local research teams. The study coordinator (AM) and the senior researchers trained the local teams.

14. How has the project contributed to improvements in local infrastructure?

According to local situational analyses based on the baseline data collected by the study, UNICEF funded structural changes in some maternity hospitals, equipment and the purchase of supplies. In all three countries, there were substantial investments in the participating centres.

15. What safeguarding procedures were used to protect local study participants and researchers?

Local ethics committees reviewed and approved the study, and informed consent was obtained from participants before data collection.
